# Diagnostic efficiency of whole-body ^18^F-FDG PET/MRI, MRI alone, and SUV and ADC values in staging of primary uterine cervical cancer

**DOI:** 10.1186/s40644-020-00372-5

**Published:** 2021-01-22

**Authors:** Aida Steiner, Sara Narva, Irina Rinta-Kiikka, Sakari Hietanen, Johanna Hynninen, Johanna Virtanen

**Affiliations:** 1 Department of Radiology, Turku University Hospital and University of Turku, PO Box 52, 20521 Turku, Finland; 2 Department of Radiology, Brigham and Women’s Hospital, 75 Francis St, Boston, MA 02115 USA; 3 Department of Obstetrics and Gynecology, Turku University Hospital, PO Box 52, 20521 Turku, Finland; 4 Department of Radiology, Tampere University Hospital, PO Box 2000, 33521 Tampere, Finland

**Keywords:** PET/MRI, 3 T MRI, Cervical cancer, Staging, SUV, ADC

## Abstract

**Background:**

The use of PET/MRI for gynecological cancers is emerging. The purpose of this study was to assess the additional diagnostic value of PET over MRI alone in local and whole-body staging of cervical cancer, and to evaluate the benefit of standardized uptake value (SUV) and apparent diffusion coefficient (ADC) in staging.

**Methods:**

Patients with histopathologically-proven cervical cancer and whole-body ^18^F-FDG PET/MRI obtained before definitive treatment were retrospectively registered. Local tumor spread, nodal involvement, and distant metastases were evaluated using PET/MRI or MRI dataset alone. Histopathology or clinical consensus with follow-up imaging were used as reference standard. Tumor SUVmax and ADC were measured and SUVmax/ADC ratio calculated. Area under the curve (AUC) was determined to predict diagnostic performance and Mann-Whitney U test was applied for group comparisons.

**Results:**

In total, 33 patients who underwent surgery (*n* = 23) or first-line chemoradiation (*n* = 10) were included. PET/MRI resulted in higher AUC compared with MRI alone in detecting parametrial (0.89 versus 0.73), vaginal (0.85 versus 0.74), and deep cervical stromal invasion (0.96 versus 0.74), respectively. PET/MRI had higher diagnostic confidence than MRI in identifying patients with radical cone biopsy and no residual at hysterectomy (sensitivity 89% versus 44%). PET/MRI and MRI showed equal AUC for pelvic nodal staging (both 0.73), whereas AUC for distant metastases was higher using PET/MRI (0.80 versus 0.67). Tumor SUVmax/ADC ratio, but not SUVmax or ADC alone, was significantly higher in the presence of metastatic pelvic lymph nodes (*P* < 0.05).

**Conclusions:**

PET/MRI shows higher accuracy than MRI alone for determining local tumor spread and distant metastasis emphasizing the added value of PET over MRI alone in staging of cervical cancer. Tumor SUVmax/ADC ratio may predict pelvic nodal involvement.

**Supplementary Information:**

The online version contains supplementary material available at 10.1186/s40644-020-00372-5.

## Background

Cervical cancer is the most common gynecological cancer worldwide [1]. Although the incidence has been falling in developed countries with available screening, cervical cancer remains a major public health problem that is affecting particularly younger women. Approximately 30% of new cases are being diagnosed in women under the age of 40 [1, 2]. Cervical cancers are staged according to the International Federation of Gynecology and Obstetrics (FIGO) or parallel TNM system by the Union for International Cancer Control (UICC). Staging is crucial not only for predicting patient survival but also for treatment planning since surgery is the standard treatment for early stage cancers (FIGO stage ≤IIA) while chemoradiotherapy is used in more advanced stages [3].

Until recently, the FIGO staging system was based on clinical pelvic examination and did not account for nodal status. However, clinical staging is inaccurate in advanced disease [4]. Furthermore, studies were showing the superiority of MRI over clinical examination in measuring tumor size and detecting parametrial invasion (FIGO stage ≥IIB) [5, 6]. In the revised 2018 FIGO staging system, imaging is now recognized as a key component in pre-treatment evaluation [7]. Pelvic MRI and whole-body PET/CT are the methods of choice, whenever available, to assess local tumor spread and nodal or distant metastases, respectively [8].

PET/MRI combines the anatomic, functional, and metabolic information from MRI and PET and may provide a “one-stop shopping” for staging and treatment planning in patients with cervical cancer. While clinical PET/MRI was introduced already in 2011, the evidence for its indications is still emerging [9]. A recent scoping review identified a literature of only 14 publications concerning the use of PET/MRI in gynecological cancers [10], majority of which evaluated recurrence detection and restaging [11–15], or associations between quantitative SUV and ADC values [16–20]. To date, only one study has evaluated the diagnostic value of PET/MRI to that of MRI alone for initial staging in patients with primary cervical cancer [21]. However, no quantitative SUVmax or ADC parameters were included in analyses.

Aim of this study was to evaluate the diagnostic performance of PET/MRI and MRI alone in local, nodal, and distant staging of patients with primary cervical cancer prior to definitive treatment. Furthermore, the objective was to assess whether tumor SUVmax and ADC values could differentiate patients according to stage. We hypothesize that PET/MRI provides an accurate tool for initial assessment of patients with cervical cancer.

## Methods

### Patients

The institutional ethics committee approved this retrospective single-institutional cohort study and the informed consent was waived. Inclusion criteria were (1) histopathologically-proven primary uterine cervical cancer of any stage and (2) whole-body ^18^F-FDG PET/MRI performed according to the institutional clinical protocol between January 2013 and April 2016 prior to definitive treatment. At Turku University Hospital, PET/MRI has been included as part of the pretreatment staging protocol for all cervical cancer patients since January 2013. A total of 33 patients were finally included and three patients excluded due to deviating MRI protocol (no diffusion weighted imaging, DWI). Mean age was 51 years (range 24–77 years). Twenty-three patients underwent surgery and 10 patients chemoradiation as the first-line treatment, respectively. Sentinel lymph node dissection with pathologic ultrastaging was successfully performed in 18 out of 23 patients undergoing surgery. Fourteen patients had undergone loop electrosurgical excision procedure (LEEP) prior to PET/MRI and definitive treatment. Baseline patient and tumor characteristics are presented in Table 1.

**Table 1:** Baseline characteristics

Characteristic	No. of patients (%)
Histology	
Squamous cell carcinoma	22 (67%)
Adenocarcinoma	10 (30%)
Adenosquamous	1 (3%)
Grade	
Gr1	5 (15%)
Gr2	15 (46%)
Gr3	13 (39%)
LVSI	
no	11 (50%)
yes	11 (50%)
Primary treatment	
Surgery	23 (70%)
Chemoradiation	10 (30%)
Type of surgery	
Radical hysterectomy + BSO/S	17 (74%)
Total hysterectomy + S	1 (4%)
Trachelectomy	2 (9%)
BSO	3 (13%)
Type of lymphadenectomy	
Pelvic	18 (78%)
Pelvic + Para-aortic	3 (13%)
Para-aortic	1 (4%)
Sentinel node resection only	1 (4%)

*LVSI* lymphovascular space invasion, *BSO* bilateral salpingo-oophorectomy, *S* Salpingectomy

### PET/MRI

^18^F-FDG PET/MRI was performed on a hybrid 3 T Ingenuity TF PET/MRI scanner using a phased-array SENSE XL Torso coil (Philips Healthcare, Best, Netherlands) [22]. All patients fasted for at least 6 h prior to the intravenous injection of ^18^F-FDG (4 MBq/kg). Pelvic and whole-body MRI were performed first, followed by patient table rotation into the PET gantry. PET was acquired from skull base to mid-thigh in multiple bed positions (18 cm each with 9 cm overlap) for 1 min 30 s per position. Activity and time from injection were taken into account per patient when computing SUV. PET images were reconstructed using 3D iterative time of flight reconstruction in 144 × 144 matrices with 4 × 4 × 4 mm^3^ voxel size. MR-based attenuation correction (MRAC) was performed by a vendor-supplied standard 3-segment (air, soft tissue, lung) method [23]. The estimated time of MRI was 45 min including the following sequences: 1) coronal T2-weighted (T2w) of the abdomen, 2) axial fat-saturated T2w of the pelvis, 3) axial fat saturated diffusion weighted imaging (DWI) of the pelvis (b-values of 0, 50, 400, and 800 s/mm^2^), 4) focused T2w of the cervix in 3 planes, tilted according to cervical long axis (sagittal, oblique axial, and oblique coronal), 5) focused fat-saturated T1-weighted (T1w) oblique axial of the cervix before and after the intravenous contrast injection of 0.2 ml/kg (0.5 mmol/ml) gadoterate meglumine (Dotarem, Guerbet, Villepinte, France), 6) axial fat-saturated 3D T1w gradient echo of the pelvis, 7) whole-body axial T2w (*n* = 11) or T1w Dixon (water) post-contrast (*n* = 22), 8) whole-body axial attenuation correction T1w. In total, PET/MRI scanning time ranged between 60 and 73 min depending on the patient’s height. Details of MRI parameters are presented in Supplementary Table 1.

### Image analysis

PET/MRI was analyzed by a senior abdominal radiologist (JV) with more than 10 years of experience in MRI and 2 years of experience in PET fusion imaging. Review of MRI alone was performed by another senior radiologist (IRK) also with more than 10 years of experience in abdominal MRI. Analyses were performed using AW Server version 2.0 (GE Healthcare, Chicago, IL, U.S.) and Carestream PACS (Carestream Health, Rochester, NY, U.S.), respectively. Both readers were blinded to all clinical and follow-up data. For the evaluation of local tumor spread, the presence or absence of tumor invasion into deep cervical stroma (≥50% of the stromal thickness), parametrium, vagina, uterus, ovaries, bladder, rectum, and pelvic sidewall were recorded. Invasion into the bladder or rectum was reported positive when mucosal wall invasion was suspected. Tumor size was measured as the maximal cross-sectional tumor diameter visualized on T2w MRI. For nodal status, suspicious lymph nodes in pelvic (regional nodes including parametrial, obturator, presacral, and iliac), para-aortic, or other distant sites were reported. Signs of nodal involvement included increased ^18^F-FDG uptake (≥blood pool uptake), size (≥10 mm), or lymph node morphology (round, irregular, diffusion restriction). Additionally, any distant metastases (liver, lung, bone, peritoneum) were reported. Based on these evaluations using PET/MRI or MRI alone, the readers independently performed a dedicated TNM (tumor, node, metastasis) staging for each patient according to the 8th Edition of UICC TNM staging classification [3, 24].

Tumor SUVmax was obtained from the volume-of-interest manually placed on the tumor containing the pixel with highest SUV. ADC maps using b-values 0–800 s/mm^2^ were automatically computed by the vendor-supplied software. For the tumor ADC measurement, the most representative slice of b800 DWI was chosen and ROI (1.0–3.0 cm^2^) positioned to the highest and most homogenous SI area within the tumor avoiding any artefacts, vessels, and necrosis. The ROI was then duplicated to the corresponding area on ADC map and tumor ADCmean (ADC) was calculated for each patient. SUVmax/ADC ratio was calculated using ADCmean values in scale × 10^− 3^ mm^2^/s as previously described [25].

### Reference standard

Histopathological reference standard was used in patients who underwent surgery. For patients receiving chemoradiotherapy as the first-line treatment, as well as for specific sites without histological verification in operated patients, a consensus reference standard was used as previously described [14, 21]. Consensus of the local tumor extent, nodal involvement, and distant metastases was obtained in a joint meeting of two radiologists (JV and AS) and two gynecologists (SN and JH) by evaluating all available information from clinical examinations, PET/MRI, and cross-sectional follow-up imaging. Time between PET/MRI analysis and consensus evaluation was at least a year. Decrease in size and/or decrease in SUVmax under therapy were regarded as signs of malignancy, while PET-negative inconspicuous lesions with constant size were regarded as benign.

### Statistical analysis

Data characteristics are presented as median values with minimum and maximum. The sensitivity, specificity, accuracy, positive predictive value (PPV), and negative predictive value (NPV) of PET/MRI and MRI in staging were computed using crosstab. Receiver operating characteristics (ROC) curve analysis was employed to calculate area under the curve (AUC) values. Group comparisons and correlations of SUV, ADC, and SUV/ADC ratio were performed using Mann Whitney U test and Spearman’s rank-order correlation, respectively. Diagnostic performance of quantitative parameters in TNM staging was estimated using ROC-AUC analysis with Youden’s index to obtain the optimal cut-off points. Two-tailed *P* values < 0.05 were regarded as statistically significant. Statistical analyses were conducted using IBM SPSS Statistics version 25.0 for Mac (IBM Corp., Armonk, NY, USA). For calculating Youden’s index, the web-tool for ROC curve analysis (www.biosoft.hacettepe.edu.tr/easyROC/) was employed.

## Results

### Diagnostic accuracy in TNM staging and evaluating tumor size

**Fig. 1:**
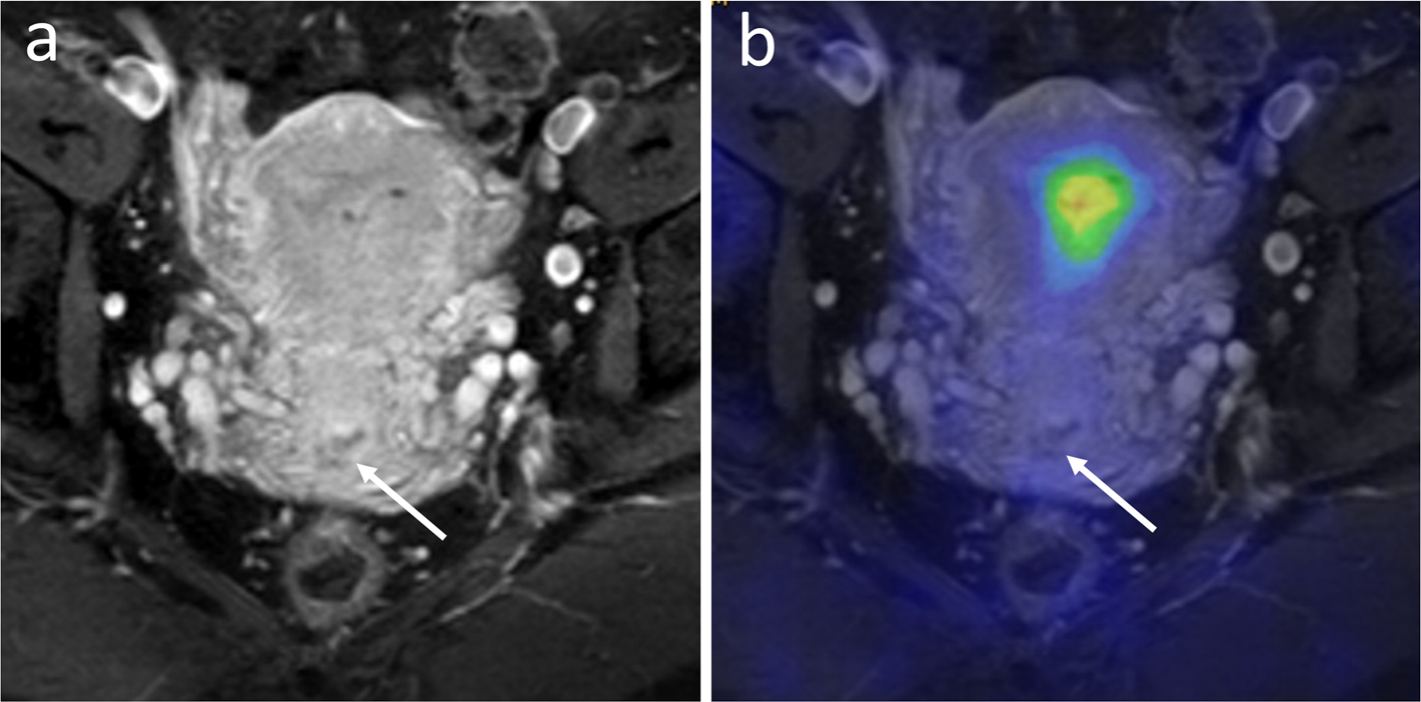
A 38-year old woman with stage T0 cervical cancer at hysterectomy that was misinterpreted as stage T1b1 on MRI. Coronal contrast enhanced T1-weighted image shows a hypoenhancing area in the cervix interpreted as a small residual following loop electrosurgical excision procedure (**a**, white arrow). PET/MRI shows no FDG uptake confirming post-procedural changes without evidence for a residual (**b**) (patient no. 9 on Table 2)

TNM staging for each patient is presented in Table 2. Based on the reference standard, T stages included T0 (*n* = 9), T1b (*n* = 10), T2a (*n* = 3), T2b (*n* = 6), T3a (*n* = 1), T3b (*n* = 1), and T4 (*n* = 3). Seventeen patients (52%) had regional lymph node metastasis (N1) and five patients (15%) had distant metastasis (M1) including liver (*n* = 1), para-aortic nodal (*n* = 3), or para-aortic and other distant nodal metastasis (*n* = 1), respectively. PET/MRI correctly identified 8/9 tumors (89%) as T0 (no evidence of tumor in the hysterectomy or trachelectomy specimen owing to radical LEEP), as opposed to 4/9 (44%) using MRI alone (Fig. 1). The median tumor size in patients undergoing hysterectomy/trachelectomy was 1.0 cm (range 0–10.0 cm) by pathology, 1.4 cm (range 0–13.0 cm) by PET/MRI, and 2.9 cm (range, 0–10.7 cm) by MRI. The estimated correlation of tumor size with pathology was higher for PET/MRI (r_s_ = 0.87, *P* < 0.001) than for MRI (r_s_ = 0.58, *P* < 0.01). Median follow-up time for all patients was 25 months (min 3 mo, max 56 mo) during which seven patients presented with recurrent disease.

**Table 2:** TNM stage by reference standard, PET/MRI, and MRI for each patient treated either with surgery or chemoradiotherapy

Patient	Reference	PET/MRI	MRI
Surgery			
1	T0 N0 M0	T0 N0 M0	T0 N0 M0
2	T0 N0 M0	T0 N0 M0	T0 N0 M0
3	T0 N0 M0	T0 N0 M0	T0 N0 M0
4	T0 N0 M0	T0 N0 M0	T1b1 N0 M0
5	T0 N0 M0	T0 N0 M0	T1b2 N0 M0
6	T0 N0 M0	T0 N0 M0	T2a1 N0 M0
7	T0 N0 M0	T1b1 N1 M0	T2b N0 M0
8	T0 N0 M0	T0 N0 M0	T0 N1 M1
9	T0 N1 M0	T0 N0 M0	T1b1 N0 M0
10	T1b1 N0 M0	T0 N0 M0	T2b N0 M1
11	T1b1 N0 M0	T1b1 N0 M0	T1b1 N0 M0
12	T1b1 N0 M0	T1b1 N0 M0	T1b1 N0 M0
13	T1b1 N1 M0	T1b1 N0 M0	T2b N0 M0
14	T1b1 N1 M0	T2a1 N1 M0	T2a1 N1 M1
15^a^	T1b1 N1 M1	T1b1 N0 M0	T2b N0 M0
16	T1b2 N0 M0	T2b N1 M0	T2b N1 M0
17	T1b2 N1 M0	T2b N0 M0	T2b N1 M0
18	T1b2 N1 M0	T2b N1 M0	T3b N1 M1
19^a^	T2a2 N1 M0	T2a2 N0 M0	T3b N1 M0
20^b^	T2b N0 M0	T1b1 N0 M0	T2b N0 M1
21	T2b N1 M0	T2b N0 M0	T2b N0 M0
22	T2b N1 M0	T2b N1 M0	T2b N1 M0
23^a^	T2b N1 M1	T2b N0 M0	T2b N0 M0
Chemoradiotherapy			
24	T1b2 N1 M0	T1b2 N1 M0	T2b N1 M1
25	T2a1 N0 M0	T2a1 N0 M0	T2b N1 Mx
26	T2a1 N1 M1	T2a1 N1 M1	T3b N1 M1
27	T2b N1 M0	T2b N1 M0	T4 N1 M0
28	T2b N1 M1	T2b N1 M1	T2b N1 M1
29	T3a N0 M0	T3a N0 M0	T3a N0 M0
30	T3b N1 M0	T3b N1 M0	T3b N1 M1
31	T4 N0 M0	T4 N0 M0	T4 N1 M0
32	T4 N1 M0	T3a N1 M0	T4 N1 M0
33	T4 N1 M1	T4 N1 M1	T4 N1 M1

*Mx* missing M staging due to technical issues; ^a^ Bilateral salpingo-oophorectomy with lymphadenectomy (no hysterectomy or trachelectomy); ^b^ No primary tumor at hysterectomy (tumor size 0 cm), however, a separate parametrial metastatic focus was present

Table 3 presents the diagnostic performance of PET/MRI and MRI alone in TNM staging. PET/MRI demonstrated higher AUC (0.89) compared with MRI alone (0.75) in determining the T stage (≥T2b), respectively. This was related to the higher specificity of PET/MRI (86%) as opposed to that of MRI alone (50%). For the determination of N stage, PET/MRI and MRI performed equally, both with an AUC of 0.73. For M staging, PET/MRI again showed higher AUC (0.80) than MRI alone (0.67).

**Table 3:** Patient-based analysis on the diagnostic accuracy of PET/MRI and MRI in determining TNM stage. Positive T stage was regarded as ≥T2b

Stage	PET/MRI						MRI					
	Sens	Spec	Acc	PPV	NPV	AUC (95% CI)	Sens	Spec	Acc	PPV	NPV	AUC (95% CI)
T stage (≥T2b 11/33)	91%	86%	88%	77%	95%	0.89 (0.76–1.00)	100%	50%	67%	50%	100%	0.75 (0.59–0.91)
N stage (N1 17/33)	59%	88%	73%	83%	67%	0.73 (0.56–0.91)	71%	75%	73%	75%	71%	0.73 (0.55–0.91)
M stage (M1 5/33)	60%	100%	94%	100%	93%	0.80 (0.53–1.00)	60%	74%	72%	30%	91%	0.67 (0.40–0.94)

*Sens* sensitivity, *Spec* specificity, *Acc* accuracy, *PPV* positive predictive value, *NPV* negative predictive value, *AUC* area under the curve, *CI* confidence interval

### Diagnostic accuracy on regional level

**Fig. 2:**
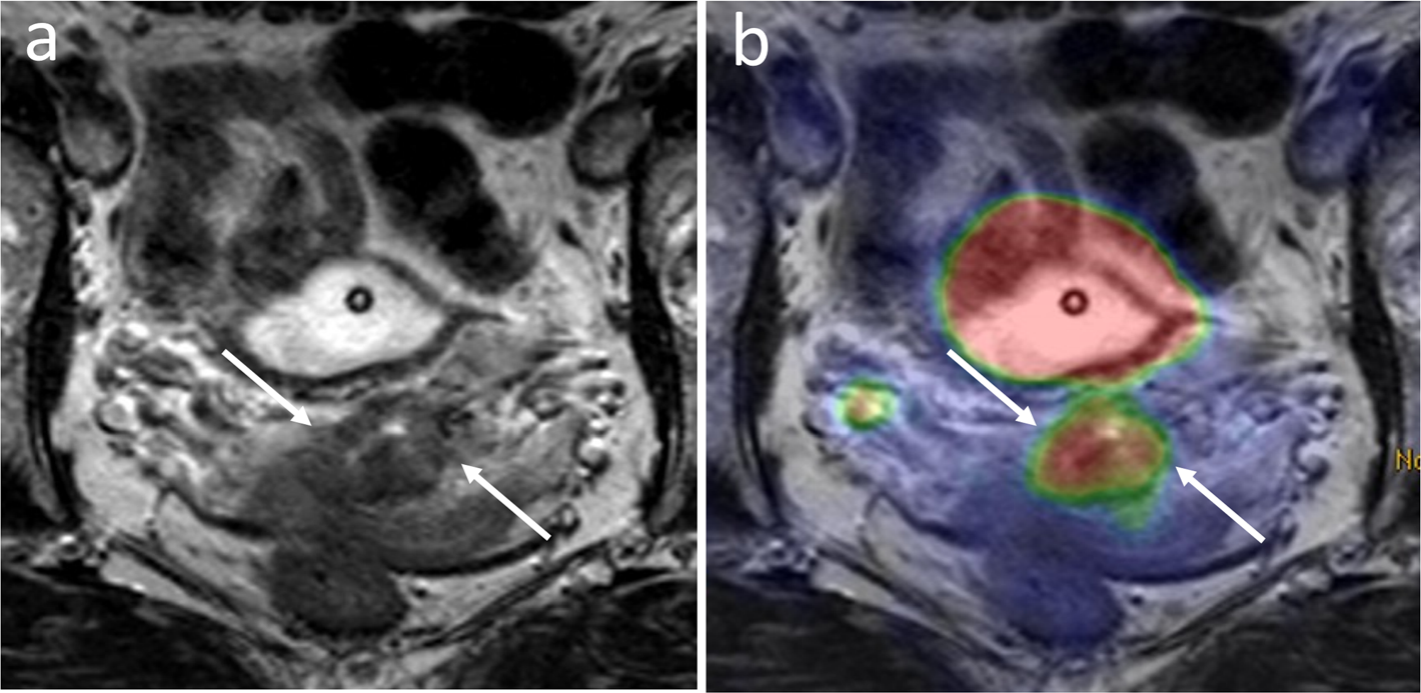
A 48-year old woman with stage T1b1 cervical cancer that was misinterpreted as stage T2b on MRI. Axial T2-weighted image shows the cervical cancer with some irregularity of the stromal rim (**a**, white arrows) interpreted as parametrial invasion by MRI. PET/MRI shows the tumor confined to the cervix with no FDG uptake in areas of stromal irregularities (**b**). The patient underwent surgical staging including parametrial biopsies and sentinel node dissection with ultrastaging, followed by curative chemoradiation (patient no. 15 on Table 2)

**Fig. 3:**
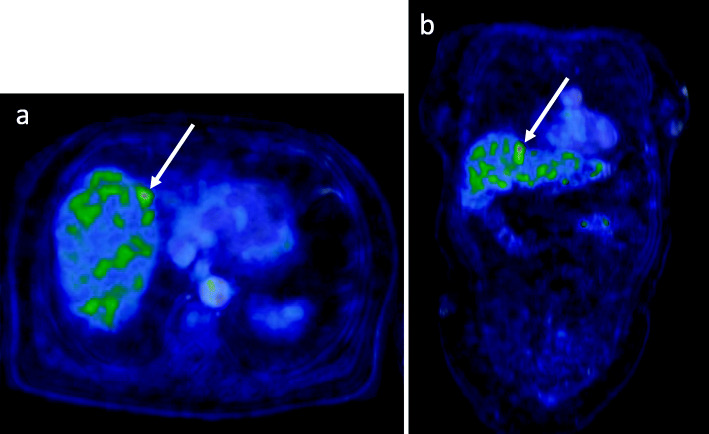
A 60-year old woman with a locally advanced disease and a small FDG avid liver metastasis in segment IV (white arrows). Whole-body PET/MRI images (with T1-weighted 3D water Dixon MRI) in axial (**a**) and coronal (**b**) plane

On a regional level, PET/MRI showed higher AUC owing to higher specificity than MRI alone in the detection of deep stromal, vaginal, and parametrial invasion, respectively (Table 4). For parametrial invasion, which is a key determinant for treatment planning, AUC was 0.89 with PET/MRI and 0.73 using MRI alone (Fig. 2). Ovaries were involved in three patients, all detected by PET/MRI but only one by MRI. Tumor extension to pelvic sidewall in two patients was detected by both PET/MRI and MRI. Bladder or rectal invasion was present in three patients. While MRI detected all three and PET/MRI two out of three, MRI diagnosed one false positive rectal invasion resulting in 97% accuracy for both modalities. In the detection of regional nodal involvement, no major difference in diagnostic performance between PET/MRI and MRI was observed (Table 4). Sensitivity for detecting nodal involvement in the left pelvic region was low both for PET/MRI and MRI. Of the seven metastases in the left pelvis that were missed by PET/MRI, four (57%) were detected by sentinel lymph node dissection and ultrastaging with a nodal size of 5 mm or smaller when reported. Corresponding number of missed metastatic nodes detected by ultrastaging in the right pelvis was 2/4 (50%). Para-aortic nodal involvement was identified in four patients. PET/MRI and MRI both failed in detecting 2/4 of these para-aortic nodes, both of which were confirmed metastatic by histology. Figure 3 presents PET/MRI of the patient with a liver metastasis.

**Table 4:** Diagnostic accuracy of PET/MRI and MRI for the detection of local tumor invasion and lymph node metastasis on a regional level

Regions	PET/MRI						MRI					
	Sens	Spec	Acc	PPV	NPV	AUC (95% CI)	Sens	Spec	Acc	PPV	NPV	AUC (95% CI)
Local invasion												
Deep stromal ^a^ (positive 9/20)	100%	91%	95%	90%	100%	0.96 (0.85–1.00)	89%	60%	74%	67%	86%	0.74 (0.51–0.98)
Vaginal (positive 12/33)	75%	95%	88%	90%	87%	0.85 (0.69–1.00)	100%	48%	67%	52%	100%	0.74 (0.57–0.91)
Parametrial (positive 10/33)	90%	87%	88%	75%	95%	0.89 (0.75–1.00)	100%	46%	63%	46%	100%	0.73 (0.56–0.90)
Lymph node metastasis												
Pelvic right (LNM in 12/33)	67%	91%	82%	80%	83%	0.79 (0.61–0.97)	75%	71%	73%	60%	83%	0.73 (0.55–0.92)
Pelvic left (LNM in 12/33)	42%	91%	73%	71%	73%	0.66 (0.46–0.87)	58%	91%	79%	78%	79%	0.74 (0.55–0.94)
Para-aortic (LNM in 4/33)	50%	100%	94%	100%	94%	0.75 (0.43–1.00)	50%	96%	91%	67%	93%	0.73 (0.41–1.00)

*Sens* sensitivity, *Spec* specificity, *Acc* accuracy, *PPV* positive predictive value, *NPV* negative predictive value, *AUC* area under the curve, *CI* confidence interval, *LNM* lymph node metastasis

^a^ ≥ 50% of the cervical stroma thickness; evaluated only in patients with histological reference

### Value of tumor SUVmax, ADC, and SUVmax/ADC ratio in TNM staging

Median tumor SUVmax was 9.6 (range 4.7–19.8), ADC 903 mm^2^/s (range 576–1054 mm^2^/s), and SUVmax/ADC ratio 11.8 (range 6.4–24.2), respectively. Eleven tumors were excluded from SUVmax and ADC measurements due to undetectable tumor at histology and/or PET/MRI. Two ADC measurements were additionally excluded due to DWI artefacts. No significant difference was observed in tumor SUVmax, ADC, or the ratio according to tumor histology (SCC versus adenocarcinoma) or grade (grade 1 or 2 versus grade 3, data not shown). There was a positive correlation between SUVmax and tumor size (r_s_ = 0.55, *P* = 0.008), while no correlation between ADC and tumor size was noted (*P* = 0.81). There was no correlation between SUVmax and ADC values (*P* = 0.93).

**Fig. 4:**
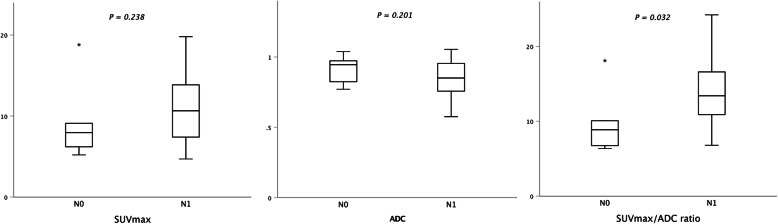
SUVmax/ADC ratio was significantly higher (*P* < 0.05) among patients with metastatic pelvic lymph nodes (stage N1) compared with patients without regional nodal involvement (stage N0). The difference was not significant when using SUVmax or ADC alone

SUVmax/ADC ratio of the tumor was significantly higher in patients with pelvic lymph node metastasis (median 13.4, range 6.8–24.2) compared with patients without pelvic nodal involvement (median 8.9, range 6.4–18.1; *P* = 0.032) as presented in Fig. 4. The difference was not significant when employing SUVmax (*P* = 0.238) or ADC (*P* = 0.201) alone. SUVmax, ADC, and the ratio were not significantly different between stage ≤T2a versus ≥T2b, or stage M0 versus M1, respectively (all *P* > 0.05).

Compared with using SUVmax or ADC alone, SUVmax/ADC ratio resulted in higher AUC for distinguishing stage N1 and M1 (Table 5). The optimal cut-off value of the ratio for N stage was 10.2. Tumor SUVmax/ADC of ≥10.2 had a sensitivity of 86% and specificity of 83% for differentiating patients with pelvic nodal metastasis (N1). For stage M1 the cut-off value was ≥12.8 yielding a sensitivity and specificity of 75 and 69%, respectively. Inverse ADC values (subtracted from 1.1) were used for T stage AUC analysis due to higher values with ≥T2b (median 0.95mm^2^/s versus 0.88mm^2^/s, *P* = 0.29).

**Table 5:** Diagnostic performance of SUVmax, ADC, and SUVmax/ADC ratio for distinguishing TNM stages. Positive T stage was regarded as ≥T2b

Stage	SUVmax	ADC	SUVmax/ADC ratio
	AUC (95% CI)	AUC (95% CI)	AUC (95% CI)
T stage	0.73 (0.52–0.95)	0.64 (0.38–0.90)	0.69 (0.44–0.93)
N stage	0.67 (0.39–0.94)	0.69 (0.44–0.93)	0.81 (0.57–1.00)
M stage	0.64 (0.37–0.90)	0.75 (0.51–1.00)	0.77 (0.56–0.97)

*AUC* area under the curve, *CI* confidence interval

## Discussion

Our aim was to assess the diagnostic efficiency of whole-body PET/MRI, MRI alone, and SUVmax and ADC values in pre-treatment staging of cervical cancer. We found that PET/MRI was superior to MRI alone for determining parametrial invasion. While MRI overestimated both the tumor size and local invasion, our results suggest that PET offers additional value compared with MRI alone in local staging and the selection of optimal treatment. Furthermore, tumor SUVmax/ADC ratio was higher in patients with metastatic pelvic lymph nodes indicating the potential predictive power of SUVmax/ADC ratio on determining nodal status.

Only one study has previously compared the diagnostic performance of PET/MRI to MRI alone in women with primary cervical cancer [21]. Sarabhai et al. performed dedicated TNM staging for 53 patients with cervical cancer and concluded that both PET/MRI and MRI equally identified the T stage (85% vs. 87%), whereas PET/MRI had higher accuracy for detecting nodal (87% vs. 77%) and distant metastases (91% vs. 83%), respectively. While our results also indicated higher accuracy with PET/MRI for detecting distant metastases (94% vs. 72%), we observed a distinct advantage of PET/MRI over MRI for determining local tumor spread with AUC values of 0.85 vs. 0.74 for vaginal, and 0.89 vs. 0.73 for parametrial invasion, respectively. Parametrial involvement is one of the most important aspects in pretreatment evaluation and should be diagnosed with high specificity since curative surgery is usually precluded if parametrial invasion is suspected [3]. MRI with DWI, also included in our protocol, is regarded a sensitive imaging method for parametrial involvement [8, 26, 27]. False-positive parametrial involvement by MRI, however, have been reported with larger tumor size and co-occurring infection [7]. Our results indicate that PET/MRI may offer higher diagnostic confidence for the detection of parametrial involvement by increasing the specificity compared to MRI alone.

PET/MRI showed improved performance compared with MRI in determining deep stromal invasion, tumor size, and in identifying patients with no residual tumor at final histology. All of these aspects are crucial when considering fertility-sparing surgery in women with early stage disease. Even without fertility sparing, however, it has been debated whether the currently established radical hysterectomy constitutes overtreatment in some patients [3]. A large randomised trial is currently ongoing to compare simple hysterectomy with radical hysterectomy in patients with cervix-confined disease with no deep stromal invasion on MRI [28]. Moreover, tumor size and the depth of stromal invasion have prognostic implications as they correlate with the risk of nodal metastases [29, 30]. Although MRI contributes anatomical details of the cervix, it may overestimate the tumor size due to post-procedure (LEEP) changes and tumor edema misinterpreted as tumor tissue. Combining the modality with PET results in higher diagnostic confidence that may be of value in early stage cancer when less radical surgery is considered. To our knowledge, evaluations of stromal invasion and tumor size agreements between PET/MRI and MRI in cervical cancer have not been reported before.

In our study, the diagnostic efficiency for detecting metastatic regional lymph nodes was equal using PET/MRI or MRI. The low sensitivity for both modalities particularly in the left pelvic region may be related with the small size of the metastatic nodes detected by sentinel node dissection and pathologic ultrastaging that is used in our hospital to allow for the detection of micrometastases [31]. Metastatic sentinel nodes missed by PET/MRI were 5 mm or smaller (when reported by pathology), emphasizing the limitation of our ability to radiographically detect small metastatic lymph nodes. The MRAC segmentation used in our PET/MRI scanner may provide another explanation for the low sensitivity. Accurate AC is required to account for the photon attenuation in tissues in order to obtain precise PET image quantification. The MRAC segmentation, however, does not delineate and take dense bone tissue into consideration, which has been shown to produce PET image bias and underestimation of SUV values in lesions that are detected close to bony structures, such as lymph nodes within the bony pelvis [32, 33]. Nevertheless, the sensitivity and specificity of PET/MRI for detecting metastatic pelvic lymph nodes in our study was 59 and 88%, respectively, which is in the range of those reported for PET/CT in patients with early stage cervical cancer (sensitivity 53–73%, specificity 90–97%) [34].

No lung metastases were detected in our cohort possibly related to early detection and the tumor being confined to cervix in majority of cases (≤T1b *n* = 19/33). In previous studies, PET/MRI and PET/CT have performed equally in the detection of pulmonary lesions ≥10 mm, while PET/MRI has shown limited ability in detecting lung lesions smaller than 10 mm [35, 36]. Even though the clinical impact of this finding is controversial as most small pulmonary nodules are known to be benign even in patients with known primary malignancy [37], caution should be used when interpreting PET/MRI for pulmonary metastasis. Fortunately, new improved MR techniques that can provide high-resolution structural information for the lung are reckoned to overcome this challenge for detecting subcentimeter lung nodules [38].

Although the comparison between PET/MRI and separately acquired PET/CT and MRI for preoperative staging in cervical cancer was not in the scope of this study, our results are similar to those using fused PET/MRI showing diagnostic accuracy of 83% for T staging (in our study 88% using PET/MRI) [39] and sensitivity of 54% and specificity of 93% for detecting nodal metastasis (in our study 59% and 88%, respectively) [40]. Compared with fused PET/MRI obtained from separate PET/CT and MRI scans, however, PET/MRI has several benefits including more than 50% lower effective radiation dose and lower risk for misregistration of PET and MR images [41].

Tumor SUVmax/ADC ratio, but not SUVmax or ADC alone, showed potential in differentiating patients with pelvic nodal involvement, which is a novel finding in cervical cancer. The ratio of SUVmax/ADC was first introduced in breast tumors where the ratio improved the categorization of lesions as benign or malignant [25]. In our study, the cutoff value of ≥10.2 resulted in a sensitivity of 86% and specificity of 83% for differentiating patients with stage N1. The benefit of using quantitative values from integrated PET/MRI for staging cervical cancer was also discovered in a recent study demonstrating the strongest prediction for pelvic nodal metastasis when tumor total lesion glycolysis (volumetric parameter derived from FDG-PET) and D_min_ (diffusion parameter derived from intravoxel incoherent motion MRI) were combined [42]. Given the challenges for detecting small metastatic nodes, the high occurrence (up to 34%) of unsuspected nodal involvement in surgery [43, 44], and the fact that nodal metastasis is the most powerful predictor for poor outcome in early stage disease [45], new indicators for predicting nodal status in patients with cervical cancer are needed. Using SUV and ADC together may improve the staging, however, larger studies are warranted.

Our study has several limitations. First, it is a retrospective study and not all factors could be controlled for, although all examinations were performed using the same PET/MRI scanner. Also, with retrospective setup a sufficient follow-up time was guaranteed to establish a reference standard for patients undergoing chemoradiation without histopathological staging. Second, the number of patients was small; a limitation also associated with other studies using PET/MRI in gynecological cancer. Third, two readers evaluated PET/MRI and MRI scans separately. Although this prevents evaluation of interobserver variability, it corresponds to routine clinical practice where experienced readers specialized in abdominal MRI and hybrid imaging report the majority of the studies per institution. Fourth, ADC values were not analyzed from the whole-tumor volume, but with ROI based mean ADC values. However, this can be considered as a robust method and used also in a clinical setting.

## Conclusions

PET/MRI is beneficial specifically in locoregional staging with higher diagnostic confidence for determining tumor size and local invasive spread compared with MRI. Furthermore, tumor SUVmax/ADC ratio was found to be a potential predictor of pelvic nodal involvement, encouraging the use of SUV and ADC together as a biparametric tool in further PET/MRI studies.

## Supplementary Information

**Additional file 1: Supplementary Table 1.** Parameters for diagnostic MR sequences.

## Data Availability

The aggregated results obtained during the current study are available from the corresponding author on reasonable request.

## Abbreviations

FDG: Fluorodeoxyglucose
PET: Positron emission tomography
MRI: Magnetic resonance imaging
SUV: Standardized uptake value
ADC: Apparent diffusion coefficient
DWI: Diffusion weighted imaging
MRAC: MR-based attenuation correction
T2w: T2-weighted
T1w: T1-weighted
FIGO: International Federation of Gynecology and Obstetrics
LEEP: Loop electrosurgical excision procedure
AUC: Area under the curve
PPV: Positive predictive value
NPV: Negative predictive value
ROC: Receiver operating characteristics
