# Supplementary material for: Diagnostic efficiency of whole-body 18F-FDG PET/MRI, MRI alone, and SUV and ADC values in staging of primary uterine cervical cancer
Source: Cancer Imaging. 2021 Jan 22;21:16. doi: 10.1186/s40644-020-00372-5 (PMC7821517; doi:10.1186/s40644-020-00372-5)
Supplement: Supplementary file 1 — Additional file 1: Supplementary Table 1. Parameters for diagnostic MR sequences. [file 40644_2020_372_MOESM1_ESM.docx]

**Supplementary Table 1** Parameters for diagnostic MR sequences

| Sequence | Anatomical coverage | Orientation | Slice (mm) | TR (ms) | TE (ms) | Matrix | FOV (mm) |
| --- | --- | --- | --- | --- | --- | --- | --- |
| T2w TSE | Renal hilum to upper thigh | coronal | 4 | 1250 | 80 | 288×275 | 375×443 |
| T2w SPAIR | Whole pelvis | axial | 6 | 1250 | 70 | 284×186 | 400×299 |
| EPI DWI (b-values 0,50,400,800 s/mm^2^) | Whole pelvis | axial | 5 | 2191 | 90 | 124×100 | 375×302 |
| T2w TSE | Small FOV pelvis | sagittal | 3 | 4142 | 90 | 224×199 | 180×199 |
| T2w TSE | Small FOV pelvis | oblique axial | 3 | 4816 | 90 | 224×199 | 180×199 |
| T2w TSE | Small FOV pelvis | oblique coronal | 3 | 4816 | 90 | 224×199 | 180×199 |
| T1w SPIR pre- and post-contrast | Small FOV pelvis | oblique axial | 3 | 565.7 | 8 | 200×200 | 180×202 |
| 3D T1w THRIVE post-contrast | Whole pelvis | axial | 1.5 | 3.0 | 1.42 | 252×198 | 375×298 |
| 3D T1w mDixon post-contrast MobiView | Whole-body | axial | 3 | 3.1 | 1.06/1.9 | 240×126 | 420×252 |
| T2w TSE | Whole-body | axial | 6 | 1150 | 80 | 332×188 | 500×349 |
| Attenuation for PET | Whole-body | axial | 6 | 4.1 | 2.3 | 200×200 | 600×600 |

TR, repetition time; TE, echo time; FOV, field of view; T2w, T2-weighted; TSE, turbo spin echo; SPAIR, spectral attenuated inversion recovery; EPI DWI, diffusion-weighted echo-planar imaging; T1w, T1-weighted; SPIR, spectral presaturation with inversion recovery; 3D, 3-dimensional; THRIVE, T1-weighted high resolution isotropic volume examination; mDixon MobiView, multi-point Dixon (water) fused into one stack of images using Philips MobiView software
